# Investigation of the Difference in the Pulse Current in the Double Pulsed Gas Metal Arc Welding of Aluminum Alloys

**DOI:** 10.3390/ma15072513

**Published:** 2022-03-29

**Authors:** Li Jin, Yuqing Yang, Ping Yao, Wenshi Chen, Zhiqiu Qian, Jiaxiang Xue

**Affiliations:** 1 School of Mechatronics Engineering, Guizhou Minzu University, Guiyang 550025, China; jinli8756@163.com (L.J.); yyq18311716050@163.com (Y.Y.); chenws200132@163.com (W.C.); qzq1969976438@163.com (Z.Q.); 2 College of Electromechanical Engineering, Guangdong Polytechnic Normal University, Guangzhou 510635, China; 3 School of Mechanical and Automotive Engineering, South China University of Technology, Guangzhou 510650, China; mejiaxue@scut.edu.cn

**Keywords:** current waveform, DP-GMAW, aluminum alloy, basic welding parameters, welding stability, mechanical properties, pores

## Abstract

In this paper, a double pulse gas metal arc welding (DP-GMAW) for an AA6061-T6 aluminum alloy based on fewer basic welding parameters than the traditional DP-GMAW is proposed. This study compared the difference in pulse base currents ($\Delta I_{b}$) and the difference in the pulse peak currents ($\Delta I_{p}$) by analyzing the electrical signal and morphology properties of welded samples. The results indicated that changing $\Delta I_{p}$ caused welding defects or even welding failure easily. The welding stability after changing $\Delta I_{b}$ was much better than that after changing $\Delta I_{p}$. The individual fish-scale width of the weld joint remained unchanged when $\;\Delta I_{b}$ was at different values. In addition, the average absorbed work, tensile strength, yield strength and elongation of the weld joints obtained by different $\Delta I_{b}$ values reached 31.1%, 60.2%, 52.9% and 37.9% of the base metal, respectively.

## 1. Introduction

Double pulse gas metal arc welding (DP-GMAW) is an efficient and novel welding technology developed on the basis of conventional pulse gas metal arc welding (P-GMAW) for the aluminum alloy [1,2]. DP-GMAW is extensively used in automobile, vessel, high-speed railway, aircraft and other industrial fields due to its special advantages, such as a beautiful weld surface, low porosity, fine grain structure and little crack incidence [3,4,5]. If the welding parameters are set properly, a high welding efficiency of DP-GMAW can be achieved under the premise of ensuring welding quality [6]. Therefore, DP-GMAW is one of the best solutions for aluminum alloy welding and it has gradually become a hot spot in the welding field [7,8].

However, when the DP-GMAW of an aluminum alloy is carried out, there are two important problems which should be faced and solved. On the one hand, the welding heat of aluminum alloy diffuses rapidly, which can easily lead to deformation and defects. On the other hand, the DP-GMAW has more welding parameters than P-GMAW, because the DP-GMAW is composed of two sets of P-GMAW that periodically alternate with each other. If the welding parameters are not well matched, it can cause a sharp deterioration in the welding effect in DP-GMAW. Therefore, reasonably matching various welding parameters is very important to form a unified adjustment expert database. Liu et al. [9,10] explored the influence of a low frequency on aluminum alloy weld formation. The results showed that the formation of aluminum alloy fish scale welds was closely related to the mutual coupling between low frequencies and droplet transfer. Jin et al. [11] investigated the effect of heat input on the properties of aluminum alloy joints in DP-GMAW by increasing the welding current. Sen et al. [12] evaluated the correlations between DP-GMAW process parameters and the bead geometry. They found that at a constant low frequency, the weld width widened and the reinforcement increased with the increase in the welding current. Wu et al. [13] made a comparative study on the microstructures and mechanical properties of weld joints produced by P-GMAW and DP-GMAW. Wu et al. [14,15] also analyzed the influence of current phase on weld seam formation and metal transfer behavior under different pulse phases in double-wire DP-GMAW. Soltani et al. [16] studied the effect of thermal frequency and current amplitude on the weldability, microstructural evolution and mechanical properties of AA7075 alloy joints welded by DP-GMAW. Liu et al. [17] believed that increasing the base current amplitude or the thermal frequency of the current effectively enhanced the oscillation of the molten pool in the DP-GMAW of an AA6061-T6 aluminum alloy. Yao et al. [18] explored the effect of a low frequency on DP-GMAW weld formation and proposed an empirical formula for the width of fish scales on the welding speed and low frequency. Mvola et al. [19] reported that the improvement in the microstructure of DP-GMAW was due to the improved heat input and energy distribution by the current waveform control. Furthermore, Wu et al. [20] compared three different thermal frequencies by changing the pulse numbers at the same heat input.

To date, there are relatively few reports on the difference in the pulse current in the DP-GMAW of an aluminum alloy. In this paper, the difference in pulse base currents and the difference in the pulse peak currents are the research objects. The influence on the difference in the pulse current on weld formation and the mechanical properties of AA6061-T6 aluminum alloy joints welded by DP-GMAW is explored.

## 2. Materials and Methods

### 2.1. Methods

**Figure 1:**
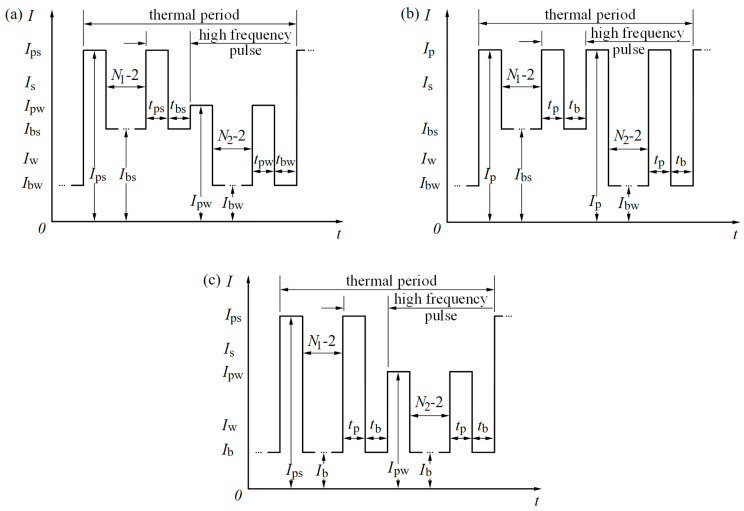
The welding current waveform of DP-GMAW: (**a**) the representative welding current waveform; (**b**) the simplified welding current waveform 1; (**c**) the simplified welding current waveform 2.

DP-GMAW is a method to achieve a welding process by selecting a suitable thermal frequency to modulate the high frequency. The representative welding current waveform of DP-GMAW is shown in Figure 1a, which contains 10 basic welding parameters in a unit current waveform cycle: the strong pulse peak current/time ($I_{\mathrm{ps}}/t_{\mathrm{ps}}$), the strong pulse base current/time ($I_{\mathrm{bs}}/t_{\mathrm{bs}}$), the weak pulse peak value current/time ($I_{\mathrm{pw}}/t_{\mathrm{pw}}$), the weak pulse base current/time ($I_{\mathrm{bw}}/t_{\mathrm{bw}}$) and the number of strong/weak pulses ($N_{1}/N_{2}$, also called thermal peak/thermal base). These basic parameters can be composed of several derived parameters, such as the thermal frequency ($f_{\mathrm{thermal}}$, also called low frequency $f_{\mathrm{low}}$), the high frequency ($f_{\mathrm{high}}$), the average current I and the strong/weak pulse group average current ($I_{s}/I_{w}$). In this article, the difference in pulse base currents and the difference in the pulse peak currents were set to $\Delta I_{b}$ and $\Delta I_{p}$. The definitions of the parameters such as $f_{\mathrm{thermal}}$ and $f_{\mathrm{high}}$ are shown in the following formulas [17].

(1) $$f_{\mathrm{thermal}}=\frac{1}{\left(t_{\mathrm{ps}}+t_{\mathrm{bs}}\right)N_{1}+\left(t_{\mathrm{pw}}+t_{\mathrm{bw}}\right)N_{2}}$$

(2) $$f_{\mathrm{high}}=\frac{1}{t_{p}+t_{b}}$$

(3) $$I=\frac{\left(I_{\mathrm{ps}}t_{\mathrm{ps}}+I_{\mathrm{bs}}t_{\mathrm{bs}}\right)N_{1}+\left(I_{\mathrm{pw}}t_{\mathrm{pw}}+I_{\mathrm{bw}}t_{\mathrm{bw}}\right)N_{2}}{\left(t_{\mathrm{ps}}+t_{\mathrm{bs}}\right)N_{1}+\left(t_{\mathrm{pw}}+t_{\mathrm{bw}}\right)N_{2}}$$

(4) $$I_{s}=\frac{I_{\mathrm{ps}}t_{\mathrm{ps}}+I_{\mathrm{bs}}t_{\mathrm{bs}}}{t_{\mathrm{ps}}+t_{\mathrm{bs}}}$$

(5) $$I_{w}=\frac{I_{\mathrm{pw}}t_{\mathrm{pw}}+I_{\mathrm{bw}}t_{\mathrm{bw}}}{t_{\mathrm{pw}}+t_{\mathrm{bw}}}$$

(6) $$\Delta I_{b}=I_{\mathrm{bs}}-I_{\mathrm{bw}}$$

(7) $$\Delta I_{p}=I_{\mathrm{ps}}-I_{\mathrm{pw}}$$

Two simplified current waveforms of DP-GMAW are shown in Figure 1b,c, respectively. In the simplified welding current waveform 1, $I_{\mathrm{ps}}=I_{\mathrm{pw}}=I_{p}$, $t_{\mathrm{ps}}=t_{\mathrm{pw}}=t_{p}$, $t_{\mathrm{bs}}=t_{\mathrm{bw}}=t_{b}$. Similarly, in the simplified welding current waveform 2, $I_{\mathrm{bs}}=I_{\mathrm{bw}}=I_{b}$, $t_{\mathrm{ps}}=t_{\mathrm{pw}}=t_{p}$, $t_{\mathrm{bs}}=t_{\mathrm{bw}}=t_{b}$. Therefore, in both Figure 1b,c, the simplified welding current waveform of DP-GMAW has only 7 basic welding parameters. The fewer basic welding parameters, the more favorable it is to build a welding expert database.

In order to achieve the ideal welding quality, the current parameters of DP-GMAW generally abide by the following rules [1,10]:

1. $I_{\mathrm{ps}}\geq I_{\mathrm{pw}}$, $I_{\mathrm{bs}}\geq I_{\mathrm{bw}}$, $I_{s}\geq I_{w}$;
2. The point ($I_{\mathrm{ps}}$, $t_{\mathrm{ps}}$) and the point ($I_{\mathrm{pw}}$, $t_{\mathrm{pw}}$) are located in the droplet transfer zone of one droplet per pulse;
3. The pulse base current $I_{b}$ is mainly used to maintain the arc combustion, and the pulse peak current $I_{p}$ is mainly used to melt the filler wire.

**Figure 2:**
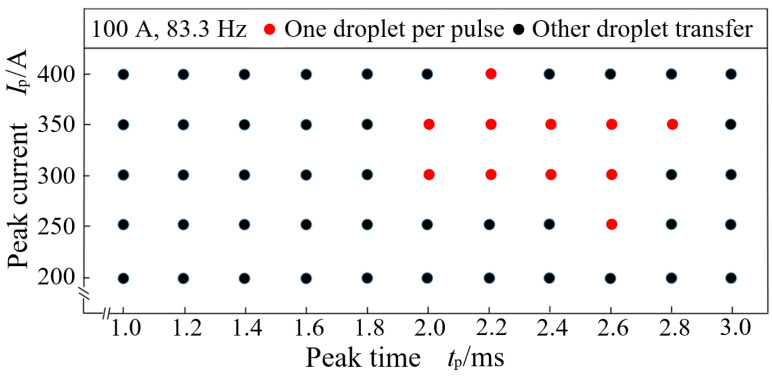
P-GMAW droplet transfer interval of one droplet per pulse at 100 A and 83.3 Hz.

In the P-GMAW process of an aluminum alloy, there are mainly three kinds of droplet transfer mode [21], i.e., one droplet per several pulses, one droplet per pulse and several droplets per pulse. Each droplet transfer mode corresponds to a specific welding arc shape [22]. Generally, one droplet per pulse is recognized as the most ideal droplet transfer mode in P-GMAW, which is the guarantee that the welded joint has a good weld formation and good mechanical properties [23,24,25,26]. Therefore, according to the morphological characteristics of the welding arc shape, one droplet per pulse zone of ER4043 welded by P-GMAW is obtained when the average welding current is 100 A and the welding frequency is 83.3 Hz, as shown in Figure 2.

### 2.2. Experiment Conditions

**Figure 3:**
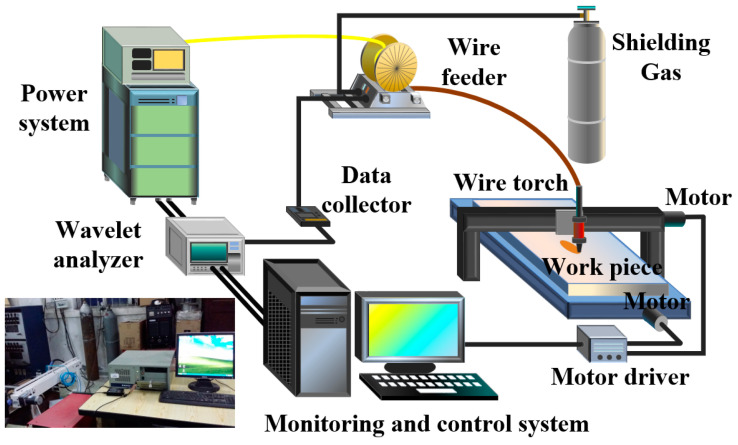
The welding system of DP-GMAW.

The welding system of DP-GMAW is shown in Figure 3 and the physical map of the welding system is located in the lower left corner of Figure 3. The power system and the wavelet analyzer were the core parts of the welding system. The DP-GMAW experiments were carried out by a self-developed power system named Pulse NBC220 (Guiyang, China). Pulse NBC220 had an Al–Mg–Si alloy welding database with the current range of 50–220 A, and its welding current waveforms included P-GMAW, DP-GMAW and SP-GMAW [27]. After the current and voltage signals were processed by the wavelet analyzer (developed by the research group, Guiyang, China), concise spectrum results and statistical analysis results were obtained by the monitoring and control system (developed by the research group, Guiyang, China), so that the stability of the DP-GMAW process could be evaluated and analyzed [17].

The base material was an AA6061-T6 aluminum alloy with dimensions of 300 mm × 60 mm × 3 mm. ER4043 with a 1.2 mm diameter was used as the filler wire. The chemical composition of the base material and the filler wire is presented in Table 1. All DP-GMAW experiments were flat plate butt welding experiments without preheating, and before each welding experiment, the starting and ending points of welding were fixed by spot welding to prevent the gap in the butt weld from changing during the welding process. The second step was to remove the stains and oxide film on the surface of the base metal with an electric wire brush, then the surface of the base metal was cleaned with acetone, and finally it was dried for welding. The shielding gas was argon with a purity of 99.99%, and the flow rate was 15 L/min. The welding speed of the DP-GMAW experiments was 40 cm/min and the thermal frequency was 5 Hz. Other welding parameters of DP-GMAW are shown in Table 2. It can be seen that the strong pulse peak current was equal to the weak pulse peak current in specimens A01–A07 and the $\Delta I_{b}$ of specimens A01–A07 was set to 10 A, 20 A, 30 A, 40 A, 50 A, 60 A and 70 A, respectively. Similarly, the strong pulse base current was equal to the weak pulse base current in specimens B01–B07 and the $\Delta I_{p}$ of specimens B01–B07 was set to 10 A, 20 A, 30 A, 40 A, 50 A, 60 A and 70 A, respectively. The values of $I_{p}$ and $t_{p}$ were located in one droplet per pulse and several droplets per pulse regions of ER4043. Specimens A01–A07 were focused on the effect of $\Delta I_{b}$, while specimens B01–B07 were focused on the effect of $\Delta I_{p}$.

**Figure 4:**
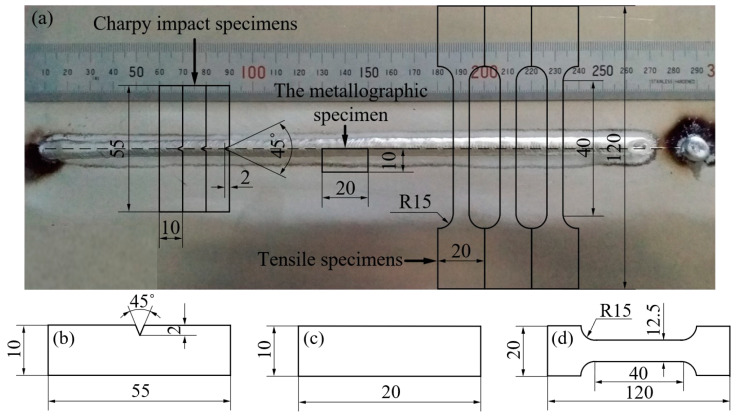
Dimensions of the test specimens (unit: mm): (**a**) extraction locations on each butt joint to obtain the test specimens, (**b**) Charpy impact specimens, (**c**) the metallographic specimen, (**d**) tensile specimens.

After all the DP-GMAW experiments in Table 2 were completed, the metallographic sample, tensile sample and Charpy impact sample were obtained from each butt weld using the electric spark cutting machine stdx600 (Huafang, Taizhou, China), as shown in Figure 4. 

The observation surface of the metallographic sample was located in the center of the weld, while the tensile samples and the Charpy impact samples were perpendicular to the center of the weld. The metallographic sample was embedded in an annular plastic mold with an epoxy resin adhesive. After the resin was cured, the metallographic sample was polished to a mirror surface after rough grinding, fine grinding and polishing. The metallographic sample was continuously corroded with Keller reagent for 35 s, then washed with deionized water and dried with a blower. Finally, the microstructure of the metallographic sample was analyzed by the stereomicroscope microscope (Bresser, Rhede, Germany) and the optical microscope (OM) (Carl Zeiss AG, Heidenheim, Germany). The equipment selected for the tensile test was an AG-IC universal electronic testing machine (Shimadzu, Kyoto, Japan). After the tensile test was finished, the morphological characteristics of the fracture were observed and analyzed by a scanning electron microscope (Hitachi, Tokyo, Japan). The Charpy impact samples were tested by a pendulum impact testing machine (Labsans, Shenzhen, China). Before the Charpy impact test, the front reinforcement and back reinforcement of the test sample need to be removed, so that the thicknesses of the whole test samples were 3 mm. The Charpy impact test was carried out according to the standard ISO 5173:2000 and the tensile test was carried out according to the standard ASTM E8.

## 3. Results and Discussion

### 3.1. The Analysis of Electrical Signals and Weld Bead Shapes

**Figure 5:**
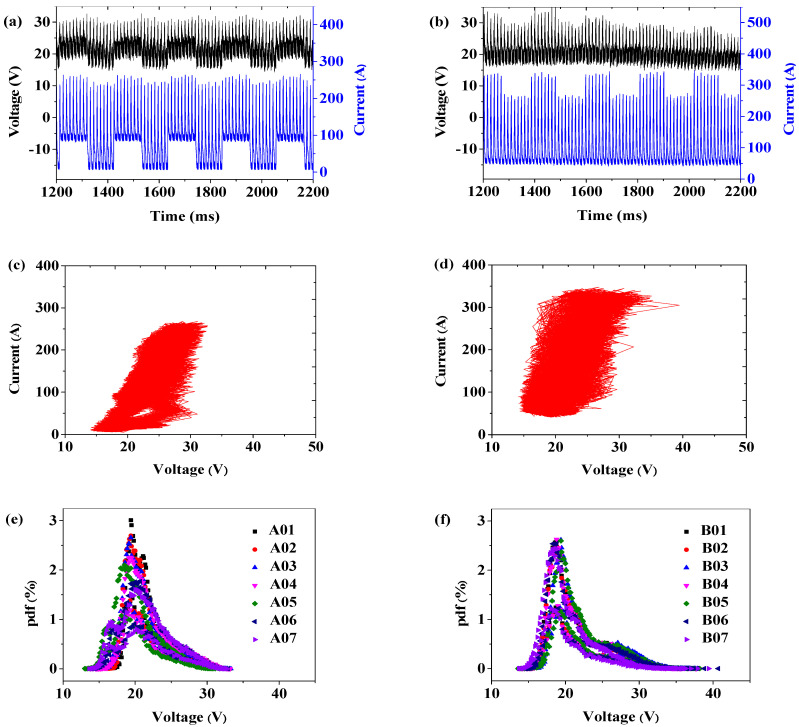
The analysis results of electrical signals: (**a**) voltage–current waveform of specimen A07; (**b**) voltage–current waveform of specimen B07; (**c**) the electrical signal statistics of specimen A07; (**d**) the electrical signal statistics of specimen B07; (**e**) welding voltage PDF results of specimens A01–A07; (**f**) welding voltage PDF results of specimens B01–A07.

The wavelet analysis results of specimen A07 (Figure 5a,c) and specimen B07 (Figure 5b,d), specimens A01–A07 (Figure 5e) and specimens B01–B07 (Figure 5f) are shown in Figure 5.

In this study, all the experiments were carried out in the same humidity and temperature environment. Figure 5a,b were voltage–current waveforms during the DP-GMAW process. In Figure 5a, the thermal pulse and thermal base of specimen A07 in both voltage waveform and current waveform were periodically generated alternately with significant DP-GMAW characteristics, which showed that the droplet transfer in the welding process had a good stability. By contrast, the current waveform in Figure 5b had a good periodicity, but the voltage waveform presented large fluctuations, indicating that the specimen B07 led to the droplet transfer of the multi-pulse one drop during the welding process. Compared with the electrical signal statistics of specimen B07 in Figure 5d, the electrical signal statistics of specimen A07 in Figure 5c were tighter and more regular and there were relatively few burrs, indicating that the DP-GMAW process of specimen A07 was more stable than that of specimen B07. Figure 5e,f show the welding voltage probability density function (pdf) results of specimens A01–A07 and specimens B01–B07, respectively. Voltage probability density has been widely used to evaluate the quality of arc welding [28,29,30,31]. It can be clearly seen that specimens A01–A07 and specimens B01–B07 had the highest voltage probability density when the welding voltage was about 19 V. The voltage probability density of specimens A01–A07 was closer and concentrated, which indicated that that specimens A01–A07 had fewer welding defects and a better welding quality. Therefore, the analysis results of the electrical signals indicate that the welding process stability of specimens A01–A07 was much better than that of specimens B01–B07 under the same welding current, low frequency and welding speed.

The weld formation of specimens A01–A07 and specimens B01–B07 is shown in Table 3. The weld bead shapes of specimens A01–A07 were well formed with few defects. In addition, there were beautiful fish scale ripples on the weld surface. When $\Delta I_{b}$ was 10 A, the surface of specimen A01 had faint fish scale ripples. The individual fish scale ripple width of specimen A01 was 1.33 mm. When $\Delta I_{b}$ was 20 A, 30 A and 40 A, the surface of specimens A02–A04 also had fish scale ripples and the clarity of the fish scale ripples increased as the $\Delta I_{b}$ increased. When $\Delta I_{b}$ was 50 A, the fish scale ripples of specimen A05 were better clarified than those of other specimens. The clarity of the fish scale ripples reduced as the $\Delta I_{b}$ increased when $\Delta I_{b}$ was 60 A and 70 A. Although specimens A02–A07 were better clarified than specimen A01, the individual fish scale ripple width of specimens A02–A07 was equal to that of the specimen A01. When $\Delta I_{p\;}$ was changed, there were irregular fish scale ripples in specimen B01 and specimen B04 and the weld appearance of specimen B01 and specimen B04 had no obvious welding defects except spatters. There were obvious arc breaks and spatters in specimen B02 and specimen B05. When $\Delta I_{p\;}$ was 30 A, 60 A and 70 A, specimen B03 and specimens B06–B07 had lots of welding defects, such as arc breaks, spatters, infusions, large drops and discontinuity of the weld. Among the above welding defects, spatters and infusion were present with specimens B01–B07. It should be emphasized that the fish scale ripples of specimens A01–A07 had two interesting phenomena: phenomenon 1 was that the clarity of the fish scale ripples first increased and then decreased with the increase in $\Delta I_{b}$. When $\Delta I_{b}$ was 50 A, the clarity of the fish scale ripples was the clearest. Phenomenon 2 was that the individual fish scale ripple width of specimens A01–A07 was equal to 1.33 mm, which had nothing to do with $\Delta I_{b}$. If 1.33 mm was divided by the thermal period, the result was approximately equal to the welding speed, as shown in the following formula:

(8) 1.33 mm÷0.2 s=6.65 mm/s=39.9 cm/min≈40 cm/min

The reason for phenomenon 1 was that the oscillation of the high-temperature liquid molten pool increased with the increase in $\Delta I_{b}$, so the clarity of the fish scale ripples of specimens A01–A04 became clearer with the increase in $\Delta I_{b}$. However, when the turbulence of the molten pool was too great, it caused the disorder of the liquid metal; therefore, the clarity of the fish scale ripples of specimens A04–A07 became more blurred with the increase in $\Delta I_{b}$. The reason for phenomenon 2 was that when the droplet transition of DP-GMAW was in one droplet per pulse, a fish scale pattern was formed in a thermal period [9,10].

Compared with specimens A01–A07, the weld formation of specimens B01–B07 was poor. There were a lot of welding defects, such as spatters, infusions, large drops and discontinuity of the weld. Combining the results of the electrical signal analysis in Figure 5 and the weld formation in Table 3, it could be considered that changing $\Delta I_{p}$ was likely to damage the welding stability and welding quality. Changing $\Delta I_{p}$ was not suitable for constructing the expert database of DP-GMAW for aluminum alloys. Therefore, the following test and analysis were only for the mode in which $\Delta I_{b}$ was changed.

### 3.2. Properties of the Metallographic Samples

The pore distribution in the weld bead center of specimens A01–A07 is shown in Table 4. Table 4 shows that the penetration depths of specimens A01–A07 were 6–7 mm, which was bigger than the thickness of the base material, indicating that specimens A01–A07 were fully penetrated.

**Figure 6:**
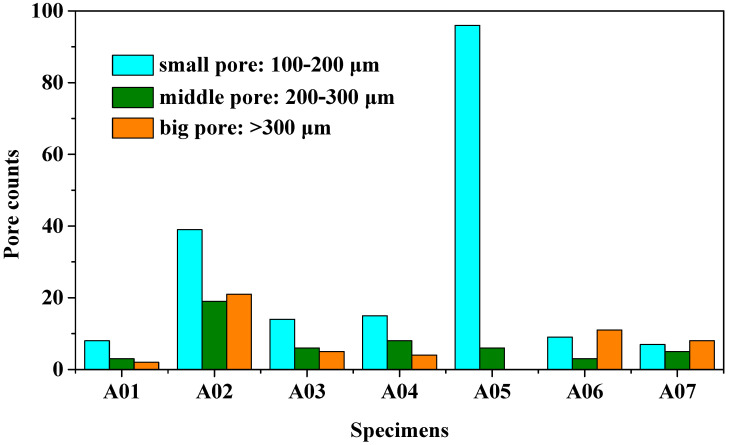
The pore distribution in the center of specimens A01–A07.

It can clearly be seen from Table 4 that there were a certain number of pores with different sizes in specimens A01–A07. Pore counts were performed on each specimen over a 20 mm representative middle section in the longitudinal direction. The pore sizes were measured using Axio Vision SE64 software. The pore statistics of specimens A01–A07 are shown in Figure 6. In this paper, pores with a diameter of 100–200 μm were defined as the small pores, the pores with a diameter of 200–300 μm and larger than 300 μm were defined as the middle pores and the big pores, respectively. Specimen A02 had the most middle pores and big pores, and specimen A05 had the greatest number of small pores and the smallest number of big pores. There were fewer pores in other specimens.

**Figure 7:**
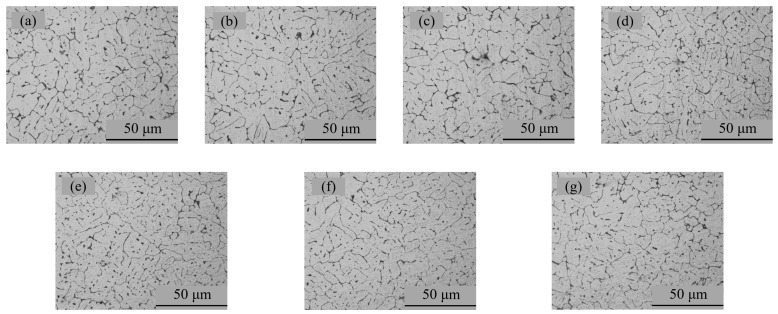
Microstructure photograph of the metallographic samples: (**a**) specimen A01; (**b**) specimen A02; (**c**) specimen A03; (**d**) specimen A04; (**e**) specimen A05; (**f**) specimen A06; (**g**) specimen A07.

The metallographic results of specimens A01–A07 are shown in Figure 7. It can be seen from Figure 7 that the weld was a typical equiaxed dendritic as-cast structure. The gray-white α(Al) interdendritic spaces were (α + Si) eutectic and a small amount of Mg_2_Si. The rapid crystallization of the molten pool caused the α(Al) dendrites to divide the liquid metal, resulting in a network of black (α + Si) eutectic crystals [32].

### 3.3. Mechanical Properties

Absorbed energy results of the base metal and specimens A01–A07 are shown in Table 5. The average absorbed energy value and the absorbed energy standard deviation of the base metal were 7.08 J and 0.14 J, respectively. The average absorbed energy of specimens A01–A07 was 2.20 J, which was 31.1% of the base metal. The average absorbed energy value of specimen A02 was 2.03 J, which was the lowest value among specimens A01–A07 and only 28.7% of the base metal. This was because specimen A02 had more middle pores and big pores than specimen A01 and specimens A03–A07. The average absorbed energy value of specimen A05 was 2.23 J, which was slightly greater than that of specimens A01–A07. However, according to the results of pore distribution in Figure 6, specimen A05 had the greatest number of small pores and the lowest number of big pores among specimens A01–A07, which did not result in a decrease in the average absorbed energy value of the weld joint. Absorption energy results of specimen A02 and specimen A05 illustrate that the average absorbed energy of the welded joint was only affected when the number and size of the pores reached a certain level. 

**Figure 8:**
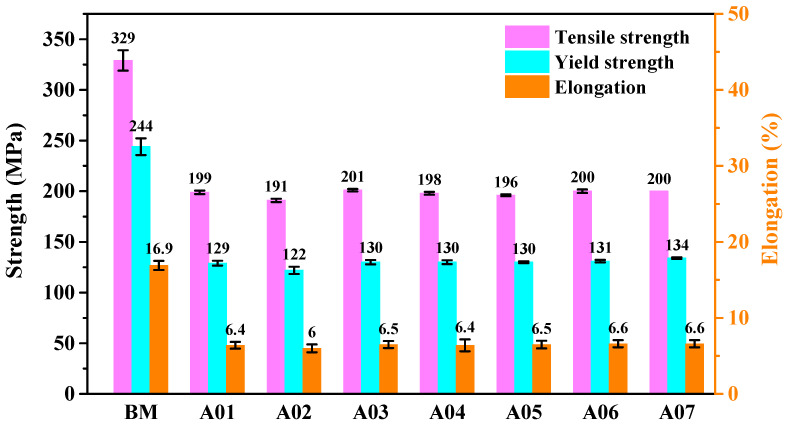
Tensile properties of the base metal and specimens A01–A07.

Figure 8 shows the tensile test results of the base metal and specimens A01–A07. Figure 8 shows that the tensile strength and the yield strength of the base material were 329 MPa and 244 MPa, respectively, and the elongation was about 16.9%.

The tensile strength, the yield strength and the elongation of specimens A01–A07 were weaker compared to the base metal. The average tensile strength, the yield strength and the elongation of specimens A01–A07 were 198 MPa, 129 MPa and 6.4%, respectively, which were 60.2%, 52.9% and 37.9% of the base metal. In particular, when $\Delta I_{b}$ was 20 A, the tensile properties of specimen A02 were the weakest among the specimens A01–A07. The tensile strength, the yield strength and the elongation of specimen A02 were 191 MPa, 122 MPa and 6.0%, respectively and only 58.1%, 50.0% and 35.5% of the base metal. This result could be due to the distribution of pores in Table 4 and Figure 6. When $\Delta I_{b}$ was 20 A, the number of middle pores and big pores in the weld was the greatest, which reduced the actual cross-sectional area of the weld and weakened the tensile properties of the joint. The tensile strength, the yield strength and the elongation of specimen A05 were 196 MPa, 130 MPa and 6.5%, respectively, which were slightly greater than those of specimens A01–A07. Therefore, the tensile properties of specimens A01–A07 were similar to the absorbed energy results. At the same time, it was noted that when $\Delta I_{b}$ was 10 A, 30 A, 40 A, 50 A, 60 A and 70 A, the weld formation was beautiful, and the absorbed energy, the yield strength, tensile strength and elongation of the welded joints were fine and changed little. These results indicate that changing $\Delta I_{b}$ was suitable for DP-GMAW with a wide matching range. Namely, $\Delta I_{b}$ could realize stable DP-GMAW welding in a large variation range; meanwhile, the joints had excellent mechanical properties.

**Figure 9:**
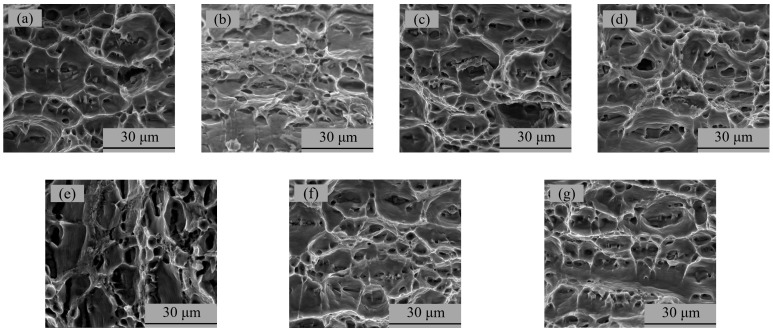
SEM photographs of the fracture surface: (**a**) specimen A01; (**b**) specimen A02; (**c**) specimen A03; (**d**) specimen A04; (**e**) specimen A05; (**f**) specimen A06; (**g**) specimen A07.

After the tensile test, the fracture morphology of specimens A01–A07 was tested by a scanning electron microscope. The specific results are shown in Figure 9. It can be seen that the SEM fracture section of the tensile sample was distributed with a large number of small and deep dimples. Meanwhile, some inclusions or second-phase particles were clearly observed at the bottom of the dimples and there was no significant difference in the size of the dimples between the different samples. The above SEM results indicate that the fracture mode of the joint was a ductile fracture.

## 4. Conclusions

The pulse amplitude is one of the key welding parameters of DP-GMAW, which significantly affects the construction efficiency and welding quality of the DP-GMAW expert database. This study compared the effects of $\Delta I_{b}$ and $\Delta I_{p}$ on weld formation, porosity and the mechanical properties of welded butt joints, and the conclusions are as follows:

(1) When the average current, the thermal frequency and welding speed were equal and the point ($\Delta I_{p}$, $t_{p}$) was located in the zone of one droplet per pulse, the DP-GMAW experiments were carried out for specimens A01–A07 and specimens B01–B07. The electrical signals of DP-GMAW were collected by the wavelet analyzer. By comparing the results of their electrical signals, it was observed that the welding process of specimens A01–A07 was more stable than that of specimens B01–B07.
(2) The weld bead shape is significantly influenced by the basic welding parameters of DP-GMAW. Specimens A01–A07 had different values of $\Delta I_{b}$ and their weld formations were wonderful, showing beautiful fish scale ripples. Specimens B01–B07 had different values of $\Delta I_{p}$, while their weld formations were much worse with many welding defects, such as spatters, infusions, large drops and discontinuity of the weld.
(3) There were some differential pores in specimens A01–A07. When $\Delta I_{b}$ was 20 A, the weld joint had the most middle pores and big pores among specimens A01–A07. The impact performance of specimen A02 was the worst, at only 28.7% of the base metal. Meanwhile, specimen A02 had the weakest tensile properties among specimens A01–A07: its tensile strength, yield strength and elongation were only 58.1%, 50% and 35.5% of the base metal.
(4) When $\Delta I_{b}$ was 10 A, 20 A, 30 A, 40 A, 50 A, 60 A and 70 A, the weld formation was beautiful and the absorbed energy, the yield strength, tensile strength and elongation of the welded joints were relatively close. The average absorbed work, maximum tensile strength, yield strength and elongation of specimens A01–A07 were 31.1%, 60.2%, 52.9% and 37.9% of the base metal, respectively.
(5) Changing $\Delta I_{p}$ can easily lead to welding instability, which is not suitable for constructing the DP-GMAW expert database of aluminum alloy. Moreover, changing $\Delta I_{b}$ can obtain beautiful weld formations and excellent joint performances, which are suitable for constructing the DP-GMAW expert database of aluminum alloys.

## Figures and Tables

**Table 1:** Chemical constituents (wt.%) of AA6061-T6 and ER4043.

Materials /Elements	Si	Fe	Cu	Mn	Ti	Mg	Al
AA6061-T6	0.52	0.25	0.01	0.96	0.01	1.0	Bal.
ER4043	6.0	<0.60	<0.30	<0.15	<0.15	<0.20	Bal.

**Table 2:** Welding parameters of DP-GMAW.

No.	*I* (A)	*I*_ps_ (A)/*t*_ps_ (ms)	*I*_bs_ (A)/*t*_bs_ (ms)	*N*_1_	*I*_pw_ (A)/*t*_pw_ (ms)	*I*_bw_ (A)/*t*_bw_ (ms)	*N*_2_	Δ*I*_b_/A	Δ*I*_p_/A
A01	100	288/2.6	53/9.4	8	288/2.6	43/9.4	8	10	0
A02	100	288/2.6	58/9.4	8	288/2.6	38/9.4	8	20	0
A03	100	288/2.6	63/9.4	8	288/2.6	33/9.4	8	30	0
A04	100	288/2.6	68/9.4	8	288/2.6	28/9.4	8	40	0
A05	100	288/2.6	73/9.4	8	288/2.6	23/9.4	8	50	0
A06	100	288/2.6	78/9.4	8	288/2.6	18/9.4	8	60	0
A07	100	288/2.6	83/9.4	8	288/2.6	13/9.4	8	70	0
B01	100	305/2.6	44.6/9.4	8	295/2.6	44.6/9.4	8	0	10
B02	100	310/2.6	44.6/9.4	8	290/2.6	44.6/9.4	8	0	20
B03	100	315/2.6	44.6/9.4	8	285/2.6	44.6/9.4	8	0	30
B04	100	320/2.6	44.6/9.4	8	280/2.6	44.6/9.4	8	0	40
B05	100	325/2.6	44.6/9.4	8	275/2.6	44.6/9.4	8	0	50
B06	100	330/2.6	44.6/9.4	8	270/2.6	44.6/9.4	8	0	60
B07	100	335/2.6	44.6/9.4	8	265/2.6	44.6/9.4	8	0	70

**Table 3:** Weld bead shape of different $\Delta I_{b}$ values and $\Delta I_{p}$ values.

No.	Weld Appearance	No.	Weld Appearance
A01	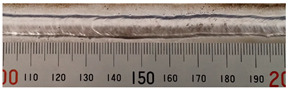	B01	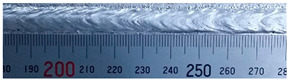
A02	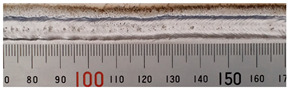	B02	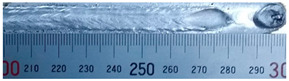
A03	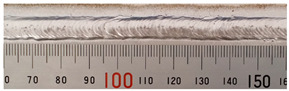	B03	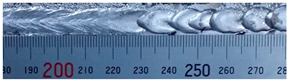
A04	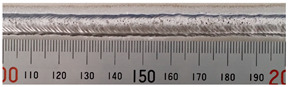	B04	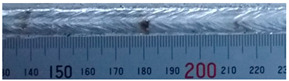
A05	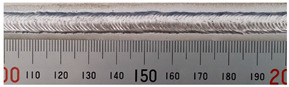	B05	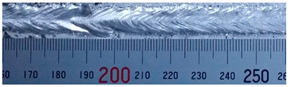
A06	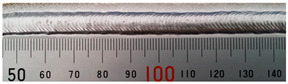	B06	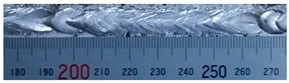
A07	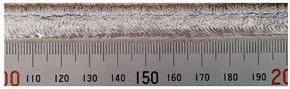	B07	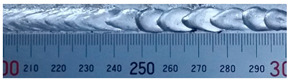

**Table 4:** Distribution of porosity on the longitudinal section of different $\Delta I_{b}$ values.

No.	The Pore Distribution in the Weld Bead Center
A01	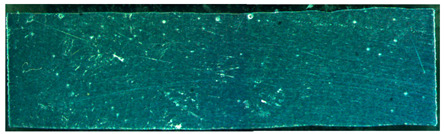
A02	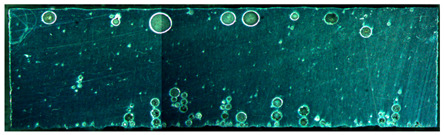
A03	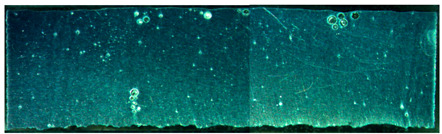
A04	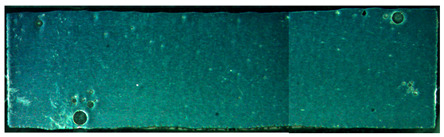
A05	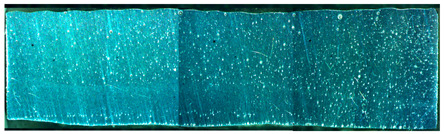
A06	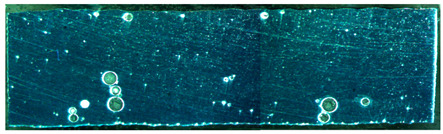
A07	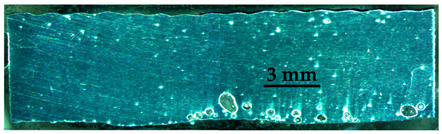

**Table 5:** Absorbed energy results of the base metal and specimens A01–A07.

No.	Absorded Energy, Akv (J)				
	1	2	3	Average Value	Standard Deviation
BM	7.25	7.00	7.00	7.08	0.12
A01	2.18	2.25	2.20	2.21	0.03
A02	2.06	1.97	2.05	2.03	0.04
A03	2.23	2.29	2.21	2.24	0.03
A04	2.42	2.03	2.25	2.23	0.16
A05	2.13	2.29	2.23	2.22	0.07
A06	2.16	2.32	2.30	2.26	0.07
A07	2.45	2.00	2.22	2.22	0.18

## Data Availability

Not applicable.
